# Influence of variation in physicochemical properties of pesticides on their adsorption isotherms and leaching behavior in egyptian clay soil

**DOI:** 10.1038/s41598-026-52244-w

**Published:** 2026-06-01

**Authors:** Mohamed R. Eshmawy, Ibrahim Abdallah, Mona A. Khorshed, Mohamed Abdelhady Kandil

**Affiliations:** 1 Ministry of Agriculture and Land Reclamation, Agricultural Research Center, Central Laboratory of Residue Analysis of Pesticides and Heavy Metals in Foods (QCAP Egypt), 7-Nadi El-Said Street, P.O. 12311, Dokki, Giza, Egypt; 2 Department of Economic Entomology and Pesticide, Faculty of Agriculture, Cairo Univ, Giza, Egypt

**Keywords:** Adsorption isotherm, Freundlich and langmuir models, Egyptian soil, Leaching behavior and tomato pesticides, Ecology, Ecology, Environmental sciences

## Abstract

The physicochemical properties of pesticides specifically solubility, volatility, and molecular structure are the primary factors determining their mobility. These factors govern their movement within the plant and their transport through the soil. The present work evaluated how variation in physicochemical properties influences the adsorption and leaching behavior of nine commonly used pesticides in tomato fields. The study includes a representative selection of major insecticides, fungicides, and herbicides to provide a comprehensive overview of their behavior in the soil. The adsorption capacity of the bulk soil was evaluated using the batch equilibration technique following two models. According to the Freundlich model, tebuconazole and difenoconazole were exhibited the highest adsorption affinity. On the contrary, the Langmuir model identified pendimethalin as having the strongest binding affinity to the soil followed by indoxacarb. Regarding the leaching experiments conducted in packed Egyptian soil columns, metalaxyl demonstrated the highest mobility, with 15% of the applied amount recovered in the leachate followed by acetamiprid. In contrast, the remaining pesticides exhibited low mobility, with the highest concentrations retained in the upper soil profile. These findings are step forward to develop management practices that mitigate the risks of pesticides, ensuring the protection of agricultural water quality and prevent groundwater contamination.

## Introduction

Pesticides have been widely used to effectively control agricultural pests to ensure the food supply for the ever-growing world population. When pesticides are introduced into the environment through spraying on crops, they may reach the soil directly or indirectly and move through the soil profile polluting water resources and adversely affect human health. Various physiochemical factors are affecting pesticides fate and behavior in the soil. Pesticides are distributed in the environment by physical processes such as sedimentation, adsorption, volatilization and leaching^1^, in which molecular weight, solubility, partition coefficient, are representing major parameters^2^. Moreover, the different substitution groups on the molecule can affect the bonding between soil and pesticides. These interactions may include van der Waals forces, ion exchange, hydrogen bonding, and covalent bonding^3^. The fate and mobility of pesticides are also influenced by other processes, such as detoxification mechanism. Hence, it is important to understand the mechanism of interaction factors that occur between pesticide molecules and the environment. Of these processes, adsorption and leaching of pesticides comprise the key factors influencing their fate and behavior in different soils.

Adsorption, is the binding between soil particles and pesticide molecules, is affected by many factors, i.e. soil type, clay minerals, organic matter ratio, pesticide properties, pH and environmental factors^4,5^. In soils that have low organic matter content, adsorption depends on the inorganic fraction, which predominantly features active compounds in the clay fraction. An increase in the clay fraction results in increased pesticide adsorption^6,7^.

Pesticide adsorption is characterized by the ratio between pesticide concentration in soil particles and soil solution, and it’s known as distribution coefficient or soil sorption coefficient (K_d_). This value is one of most important input parameters of mathematical models predicting the fate of pesticides in environments, because it is responsible for other processes such as leaching, uptake, and volatilization^8^. Thus, adsorptive capacity gives an overall estimate as to where a pesticide will be distributed in the environment.

Another crucial factor influencing pesticide behavior and characteristics in the soil is leaching. Pesticides are usually transported from soil to surface and ground water through irrigation or rainfall. Key factors affecting pesticide leaching include pesticide physicochemical properties, soil texture, organic matter content, pesticide application rate, and climatic conditions^1^. It is well known that pesticides with high water solubility exhibit lower sorption to soil particles and more likely to leach into ground water from the surface soil to different soil layers via water flow; enter other soils through surface runoff; and contaminate surface water and groundwater^9^.

Pesticide adsorption isotherm is a mathematical model that describe the relationship between the concentration of a pesticide in a solution (soil water) and the amount of that pesticide adsorbed onto a solid surface (soil particles) at a constant temperature. These isotherms are crucial for understanding properties and behavior of pesticides in the environment. The most common linear models used to describe pesticide adsorption are the Freundlich and Langmuir isotherms.

The Freundlich isotherm describes the adsorption of a substance onto a surface, assuming that the adsorption sites are heterogeneous (uneven in energy). It is more empirical and typically used when adsorption occurs on a surface with a non-uniform distribution of adsorption sites. It’s suitable for low concentrations of adsorbate^10^. While, the Langmuir isotherm assumes that adsorption occurs at specific homogeneous sites within the adsorbent, and each site can hold at most one molecule. It assumes that there is no interaction between adsorbate molecules. It is ideal for adsorption processes where the surface has a fixed number of identical sites and no further adsorption can occur once all sites are filled. It often applies to monolayer adsorption^11^. The adsorption of pesticides in soil environments is predominantly governed by the physical and chemical properties of the soil matrix, with the Langmuir and Freundlich isotherm models serving as the primary tools for quantifying these interactions. Wang et al.^12^ indicated that adsorption, desorption and mobility of propiconazole and difenoconazole on five different soils varying in organic matter and clay ratio were significantly different on each soil according to the Freundlich adsorption coefficient. Raunaq et al.^13^ also showed that, adsorption–desorption data on tebuconazole obtained from three Indian soils of different agroclimatic, regions fitted well in the Freundlich adsorption equation. The Freundlich model frequently fit for pesticide adsorption across diverse soil types including clay and alluvial soils because it accounts for the energetic heterogeneity of soil surfaces and the potential for multilayer adsorption Tolner^14^. While the Langmuir model assumes a finite number of identical sites leading to monolayer coverage, it is often used to estimate the maximum adsorption capacity Hu et al.^15^. In clay-rich soils, pesticides exhibit high adsorption due to the large surface area and high cation exchange capacity of clay minerals Ayenew and Getu^16^. In contrast, alluvial soils, often show varied adsorption profiles depending on their specific mineralogy and organic carbon content; however, the Freundlich generally indicates that adsorption on these complex surfaces is a favorable, non-linear process Wauchope et al.^17^.

Behavior of pesticides in soil under Egyptian conditions is poorly investigated. Elucidating adsorption and leaching processes as significant physical characteristic of a pesticide, allows the applicator to make better decisions about which pesticide active ingredient to use for a particular situation. Numerous studies have demonstrated that the behavior of a pesticide varies significantly depending on soil texture. For example, Arias-Estévez et al.^18^ found that pesticides with a low partition coefficient and high water solubility (such as the metalaxyl and acetamiprid in our study) are extremely prone to leaching in sandy soils with low organic matter. Consequently, if a farmer’s land consists of sandy soil, decision-makers would advise against using highly mobile active ingredients. Instead, they would recommend selecting pesticides with high log P values (such as pendimethalin or lufenuron in our study) to ensure the chemical remains in the root zone and does not contaminate the groundwater. Understanding the physicochemical properties of pesticides also helps to develop pesticide management strategy and take precautionary measures to mitigate their risk and negative effects on human health and environment.

In the present work, 9 pesticides representing different main pesticide types (5 insecticides, 3 fungicides and 1 herbicide) commonly used in tomato and belong to various families, were selected. These pesticides were chosen based on their extensive use in tomato as an economically important crop in terms of area cultivated, production and consumption rate. These nine pesticides were namley: Acetamiprid, chlorantraniliprole, emamectin benzoate, indoxacarb, lufenuron, difenoconazole, tebuconazole, metalaxyl and pendimethalin. Therefore, the aim of this study was to examine the adsorption and leaching behavior of these nine pesticides on a common alluvial Egyptian soil using the two adsorption isotherm models, Freundlich and Langmuir.

## Materials and methods

### Soil sampling

Samples of alluvial soil were collected from the Experimental farm of the Faculty of Agriculture, Giza region, Egypt. This soil has not been treated with any of the nine pesticides and quantification analysis revealed an absence of any soil pesticide residues. Soil was collected according to organization for Economic Co-operation and Development guidelines (OECD) for the testing of chemicals^19^. Samples were taken at 0- 30 cm deep, then air dried, ground and passed through a 2-mm sieve before use. The physical and chemical properties of the soil were determined at the Soil, Water and Environment Research Institute (SWERI), Agriculture Research Center, Egypt as shown in Table 1. Samples were collected from an experimental research farm with the knowledge and authorization of the farm. The sampling was conducted according to standard research practices and did not involve protected areas or cause any environmental disturbance. As this site is a research facility, no formal written permission was required for routine soil sampling activities.

**Table 1:** Chemical and physical analysis of the tested soil.

Tests	Method of analysis	Results	Range / LOD
Soil organic matter percentage	Method of soil analysis^20^	1.99%	–
PH	Soil chemical analysis^21^	8.65	0–12
Soluble Cations mg/L	ICP method^22^	Na = 13.6 mg/L	0.1 µg/L
	ICP method^22^	Mg = 5.50 mg/L	0.1 µg/L
	ICP method^22^	Ca = 9.50 mg/L	0.1 µg/L
Soluble Anions mg/L	Soil chemical analysis^21^	SO_4_ = 9.47 mg/L	5 µg/L
	Soil chemical analysis^21^	Cl = 18.5 mg/L	3.5 µg/L
	Method of soil analysis^20^	HCO_3_ = 1.0 mg/L	0.1 µg/L
Soil texture is Clay	Soil and plant analysis^23^	Clay = 56.5%	–
	Soil and plant analysis^23^	Silt = 24.0%	–
	Soil and plant analysis^23^	Sand = 19.5%	–

### Pesticides used in the experiments

The properties and main characteristics of the nine pesticides (5 insecticides, 3 fungicides and 1 herbicide) under the study are presented in Table 2.

**Table 2:** Characteristics of the tested pesticides.

Category	Common name	Chemical structure	Molecular weight g/mol	Water Solubility mgl^-1^ PH = 7	partition coefficient Log K_ow_ PH = 7	Resources of Pesticides		Target Pests
						Technical grade purity	Trade name and conc	
Insecticides	Acetamiprid		223	2950	0.8	Jiangsu Subin Agrochem. Co. 97%	Clipper 20% SL	(aphids, whiteflies and thrips)
	Chlorantraniliprole		483	0.88	2.86	Qingdao Hengning Biotech. Co. 96%	Coragen 20% SC	(armyworms and cutworms)
	Emamectin Benzoate		1008	24.0	5.0	Ningxia Taiyicin Biotech. Co. 95%	Agroclem 5.7% WG	Leaf worms and mites
Insecticides	Indoxacarb		528	0.20	4.65	Shandong Jingbo Agrochem. Tech. Co. 90%	Indoxy 30% SC	Leaf worms
	Lufenuron		511	0.046	5.12	Zhejiang Sega Science and Tech. Co. 96%	Castello 10% EC	Leafworms larvae
Fungicides	Difenoconazole		406	15.0	4.36	Yifan Biotech. Group Co. 95%	Score 25% EC	(rust, leaf spot and powdery mildew)
Fungicides	Tebuconazole		308	36.0	3.70	Shandong Huayang Pesticide Chem. Industry Co. 96%	Tebo Croos 43% SC	(rust, leaf spot and powdery mildew)
	Metalaxyl		279	8400	1.75	Zhejiang Heben Pesticide & Chem. Co. 98%	Twin MZ 72% WP	Mildew and damping off
Herbicides	Pendimethalin		281	0.33	5.40	Shandong Huayang Pesticide Chem. Industry Co. 95%	Esenda 45% CS	Annual grasses and broadleaf weeds

### Reagents and pesticides

All the tested pesticides were obtained from different companies and were used as technical grade with purity ranged from 93 to 99%. All chemicals were LC- MS analytical grade, including calcium chloride, acetonitrile, methanol, ammonium acetate, ethyl acetate, acetic acid, extraction salts (Sodium chloride and anhydrous magnesium sulfate), Buffers (Acetate buffers and citrate buffers) and purified water was prepared using a Milli-Q purification (A10 FOCN53824k). Ammonium acetate solution was prepared as a buffer solution and then its pH was adjusted to 4.5 with acetic acid.

### Apparatus

Analysis of samples was performed by High Performance Liquid Chromatography (HPLC) using an Agilent 1200 series equipped with an Agilent Eclipse Plus C18 analytical column (5 μm × 150 × 4.6 mm), a photodiode array detector (Agilent G1315C), a vacuum degasser (G1322), an autosampler (G1313), a quaternary pump (G1311A), and ChemStation for LC, Rev. A. 09.03 [1417] software. Other equipment included a Milli-Q water purification system A10 FOCN53824k, a Mettler Toledo lab balance capable of weighing to 0.1 mg, an ADWA pH meter, a HERMLE Labortechnik GmbH (Wehingen, Germany) Centrifuge Z 446 K with a centrifugal force (RCF) of 16,020 g for 10 × 50 mL tubes, a shaker, and Hirschmann micropipettes ranging from 1 to 100 µL and 100 to 1000 µL.

### Determination of equilibrium adsorption isotherm

**Fig. 1:**
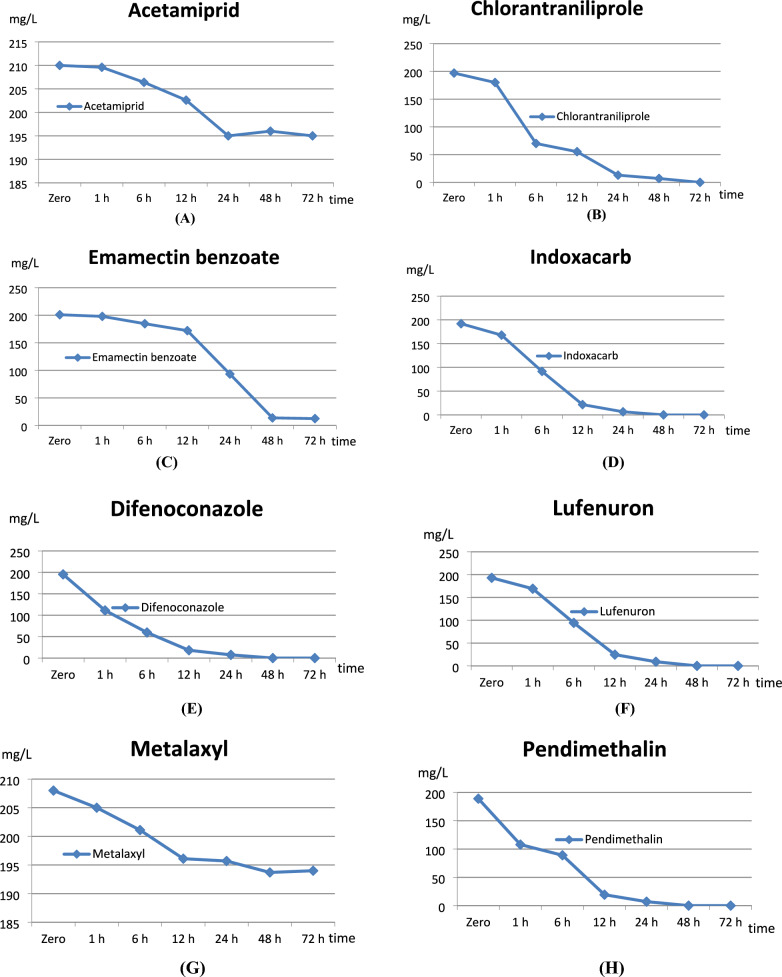

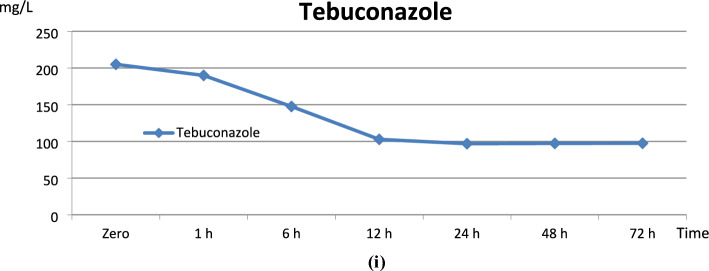
The equilibrium time for adsorption isotherm (**A**) Acetamiprid, (**B**) Chlorantraniliprole, (**C**) Emamectin benzoate, (**D**) Indoxacarb, (**E**) Difenoconazole, (**F**) Lufenuron, (**G**) Metalaxyl, (**H**) Pendimethalin, and (**I**) Tebuconazole.

Equilibrium adsorption isotherm was used to measure the capacity, surface properties, and affinity of the soil (adsorbent) for adsorbing various pesticide compounds (adsorbate). The experiment utilized 63 soil samples, each analyzed in duplicate for a total of 126 analyses with a mass ratio of pesticide solution to bulk soil of 1:25 (v/w). Experiments were conducted under laboratory conditions at 20–23 ℃. Pesticides concentrations were 200 mg/L with three replicates and prepared in 0.01 M calcium chloride to improve centrifugation and minimize cation exchange. The experiment of equilibrium intervals was 0, 1, 6, 12, 24, 48, and 72 h for each individual pesticide. The equilibrium adsorption isotherm time is shown in Fig. 1.

Adsorption isotherm experiments (Freundlich and Langmuir) were performed according to organization for Economic Co-operation and Development guidelines OECD for the testing of chemicals^19^.

### Freundlich model for adsorption isotherm

The experiments were conducted for the pesticides under the study at three different concentrations (100, 200, and 300 mg/L) with 2 replicate, based on the equilibrium time for each pesticide. The experiment was carried out in 15 mL polypropylene centrifuge tubes (0.4 g soil and 10 mL pesticide solution). A control sample (soil and 0.01 M calcium chloride) and a blank sample (pesticide solutions with no soil) were included. Throughout the experiment, the tubes were shaken with a Geno shaker at room temperature until equilibrium, based on our kinetics study. The samples were then centrifuged at 4000 rpm at 5 ℃ for 15 min. The supernatants containing the pesticide were filtered by a 0.2 µm Acrodisc and measured by HPLC.

The amount of pesticide adsorbed by solid phase at equilibrium according to Freundlich model was calculated using following Equation:

1 $$Cs=(Ci-Ce) \times \frac{V}{M}$$

where C_s_ is the concentration of pesticide adsorbed per mass of soil (mg/g), C_i_ is the initial concentration of pesticide, and C_e_ is the concentration of pesticide at equilibrium (mg/L). M_s_ is the weight of the soil (adsorbent), and V is the volume of the pesticide solution. Furthermore, the constant values of the isotherm were calculated from the slope and the intercepts of the plots using the least squares method. The adsorption isotherm is described by calculating the logarithmic form using following Equations of Freundlich:

2 $$\mathrm{log}qe=\mathrm{log}Kf+\frac{1}{n} \times \mathrm{log}Ce$$

3 $$n=\frac{1}{slope}$$

4 $$Kf= \mathrm{a}\mathrm{n}\mathrm{t}\mathrm{i} \mathrm{l}\mathrm{o}\mathrm{g} \mathrm{i}\mathrm{n}\mathrm{t}\mathrm{e}\mathrm{r}\mathrm{c}\mathrm{e}\mathrm{p}\mathrm{t}$$

where q_e_ is the concentration of pesticide adsorbed per mass of soil (mg/g), 1/n is the Freundlich adsorption exponent and K_f_ is the Freundlich adsorption coefficient. The values of n = 1/slope and K_f_ = anti log of intercept. In the Freundlich model, the R^2^ value is important because if R^2^ = 1, the model is perfect and explains 100% of the variability in the data. If R^2^ is greater than 0.95, it indicates a good fit, while R^2^ less than 0.9 shows a poor fit, demonstrating that the model does not well explain data variability. K_f_, the Freundlich constant, is a measure of the adsorbate’s attraction to the adsorbent: a higher K_f_ suggests a stronger affinity. The closer the value of n is to 1, the more favorable the adsorption as described in previous study that tested the effect of clay on the adsorption isotherm of pesticide^8^.

### Langmuir model for adsorption isotherm

The experiments were conducted in the same manner as the Freundlich experiment. The amount of pesticide adsorbed onto the solid phase at equilibrium, according to the Langmuir model, was calculated using Equation:

5 $$qe=(Ci-Ce) \times \frac{V}{M}$$

where q_e_ is the concentration of pesticide adsorbed per mass of soil (mg/g), C_i_ is the initial concentration of pesticide while C_e_ is the concentration of pesticide at equilibrium (mg/L), M is the weight of soil (adsorbent) and V is the volume of pesticide solution. Furthermore, the constant values of isotherm were calculated from the slope and the intercepts of the plots using the least squares method. The description of Langmuir adsorption isotherm is by calculating from following Equations:

6 $$qmax=\frac{1}{\mathrm{i}\mathrm{n}\mathrm{t}\mathrm{e}\mathrm{r}\mathrm{c}\mathrm{e}\mathrm{p}\mathrm{t}}$$

7 $$Kl=\frac{1}{( \mathrm{S}\mathrm{l}\mathrm{o}\mathrm{p}\mathrm{e} \mathrm{x} \mathrm{q}\mathrm{m}\mathrm{a}\mathrm{x})}$$

8 $$Rl=\frac{1}{1+ ( \mathrm{C}\mathrm{i} \mathrm{x} \mathrm{K}\mathrm{l})}$$

In the Langmuir model, the R^2^ value is important because if it is greater than 0.95, it indicates a good fit for explaining the data., when R^2^ is less than 0.9, it is considered a poor fit, and this model does not explain the variability in the data well. Additionally, the R_L_ value is crucial for describing the Langmuir model: if the R_L_ value is less than zero, adsorption is unfavorable; if the R_L_ value ranges from zero to one, adsorption is favorable; and if the R_L_ value is more than 1, adsorption is reversible as described in previous study that perform experiment of the adsorption isotherm of some pesticides on different size of clay^4^.

### Determination of the tested pesticides

#### Adsorption isotherm experiment

**Fig. 2:**
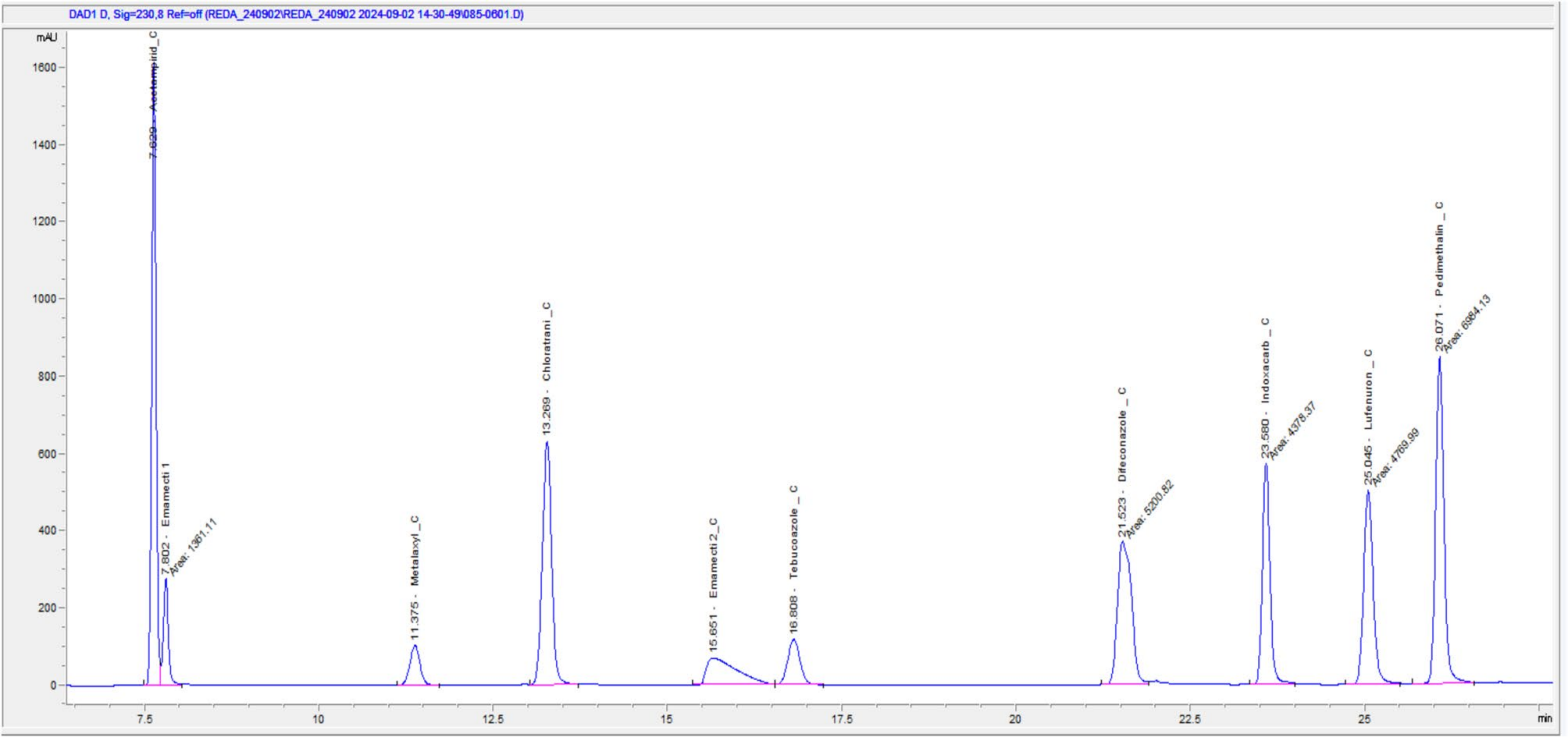
The chromatogram of tested pesticides.

Individual standard solutions of 100 mg/L for each pesticide were prepared by dilution from a 1000 mg/L stock solution in aqueous dimethyl sulfoxide (DMSO). Each pesticide was analyzed by HPLC at several wavelengths: 205, 210, 220, 230, 240, 250, and 290 nm to determine the optimal wavelength based on peak area (intensity), accuracy, and lowest interference. After selecting the best wavelength, the other wavelengths remained in the injection method to verify the accuracy of the results. A mixed standard solution of 100 mg/L containing all tested pesticides was injected. After several attempts, the spectra of the selected pesticides were separated from each other by changing the ratio of the mobile phases (ACN and phosphate) at different times. The best spectrum was chosen based on intensity and minimal interference, as shown in Fig. 2.

The analysis time for one sample or standard was 35 min. The optimal wavelengths for the pesticides are shown in the results section. A calibration curve from six points of the pesticide mixture (25, 50, 100, 200, 400, and 500 mg/L) was prepared, and the lowest correlation coefficient obtained was 0.999.

For pesticide analysis in adsorption isotherm experiment, the method were validated following the European guidelines on analytical quality control and method validation for pesticide residues in food and feed (SANTE/11,312/2021v2)^24^. There are no need for evaluation of matrix effects because our analysis were by HPLC photodiode array, so there is no signal reduction in this system like LC MS–MS. Controlling our analysis by injection calibration blank, calibration curve, standard check and then free sample blank followed by its spike to check recovery. Spiking samples were performed at three different levels (100, 200, and 400 mg/kg) in free soil samples. The evaluated parameters included linearity, precision, trueness and limit of quantification (LOQ). Linearity as the deviation of back-calculated values from expected concentrations (± 20%), Precision as the relative standard deviation (RSD, ≤ 20%), Trueness was evaluated based on the average recovery percentages obtained from fortified soil samples (Spike) with acceptable range: 70–120% and LOQ as the lowest validated spike level meeting the acceptance criteria for trueness and precision.

#### Leaching behavior of the tested pesticides

A single standard solution of 100 mg/L was injected and the best wavelength spectrum for each pesticide individually was chosen based on intensity and interference. The optimal wavelength and retention time for each individual pesticide were similar to those in the Adsorption Isotherm experiment, except for emamectin benzoate, lufenuron, and tebuconazole, which had retention times of 7.6, 6.3, and 17.2 min, respectively. The calibration curve from six points for each individual pesticide (15, 25, 50, 100, 200 and 400 mg/L) was prepared. The lowest correlation coefficient was 0.999 and the maximum analysis time was 26 min as showed in the results section.

Leaching experiments were conducted according to OECD guidelines for the testing chemicals using formulations of the tested pesticides^25^. Studies were conducted in the dark under laboratory conditions, with temperatures ranging from 18 to 25 °C, using the same soil type as in the Adsorption Isotherm experiment. Leaching experiments were done by using two replicates of soil glass columns for each pesticide (inside diameter 6 cm and length diameter 60 cm). Columns for all tested pesticides were segmented from the top into 5 parts; each part was 10 cm long to estimate the mobility potential of tested pesticides. The lower opening of the column was closed by filter paper filled with glass wool.

Each column was filled with air dried soil (< 2 mm) and the amount of soil in each column has the same average weight 900 to 920 g. The upper opening of the column was covered by filter paper to prevent soil spattering and then each column was preconditioned before the test by artificial rain (0.01 M CaCl_2_) according to OECD guidelines. Two replicates of columns were filled with soil and kept as control without pesticide addition. After the addition of tested pesticides on the top of column, the flow rate of water was selected specifically to achieve saturated flow conditions within the soil and to minimize the duration of the leaching experiment. This short timeframe helps to reduce the potential for the pesticide to degrade during the test, about 400 ml of water was added to simulate continuous flowing manner of rainfall as described by Singh et al.^26^ and also corresponding to the field capacity (70% for clay soil and 30% for sandy soil) according to and the amount of water is also added gradually in 8 stages. Finally, the leachate fractions from each column were collected and analyzed to determine the concentration of each pesticide. The columns were left to drain for a day, then divided horizontally into 10 cm sections and air-dried for 3 days in the dark, preparing them for pesticide analysis. The recovery percentage of pesticides in soil sections was calculated from the calculation:

9 $${\left(\mathrm{R}\mathrm{e}\mathrm{c}\mathrm{o}\mathrm{v}\mathrm{e}\mathrm{r}\mathrm{y} \mathrm{p}\mathrm{e}\mathrm{r}\mathrm{c}\mathrm{e}\mathrm{n}\mathrm{t}\right)}^{n}=\frac{\mathrm{F}\mathrm{o}\mathrm{u}\mathrm{n}\mathrm{d} \mathrm{C}\mathrm{o}\mathrm{n}\mathrm{c}.}{\mathrm{F}\mathrm{o}\mathrm{r}\mathrm{t}\mathrm{i}\mathrm{f}\mathrm{i}\mathrm{c}\mathrm{a}\mathrm{t}\mathrm{i}\mathrm{o}\mathrm{n} \mathrm{C}\mathrm{o}\mathrm{n}\mathrm{c}.} \times 100$$

#### Determination of pesticide concentrations in leachate samples (water)

The extraction of pesticide from leachate samples (Extraction Vacuum Manifold) by using solid phase extraction (SPE) with polymer based hydrophilic lipophilic balanced SPE cartridges (Oasis HLB) according to authors Belal et al*.,* 2016, Liu et al*.*, 2006 and Refai et al*.,* 2022^27–29^ after filtered with filter paper, the SPE cartridge was preconditioned by add 5 ml Acetonitrile (ACN) and then 5 ml methanol (MoH) and finally 10 ml Deionized water (DIW) at flow rate 15 ml/min. The leachate samples were passed through the cartridge with the same flow rate. The pesticides that adsorbed on SPE were eluted by 10 ml ACN and the elution process is carried out in two stages then injected in HPLC.

#### Determination of pesticide concentrations in soil samples

The extraction of pesticide from soil samples were done by QUECHERS method for pesticide residue analysis according to authors El- Gohary, 2014 and Saleh et al*.,* 2020^30,31^, each 10 cm of the column is considered a single soil sample. Ten grams from soil sample were weighted into the polypropylene centrifuge tube then 10 ml of 1% acetic acid was added and shaking by shaker for 2 min and ultrasonic for 1 min. 10 ml from ACN was added then the tubes were closed and shake vigorously for 2 min. Add reagent (1) (1 g sodium citrate, 0.5 g sodium hydrogen citrate sesquihydrate, 1 g sodium chloride and 4 g magnesium sulphate) and shake for 2 min and then centrifuge for 5 min with 4000 rpm at 5 ℃. The aliquot of ACN phase was taken for HPLC analysis.

For pesticide analysis in leaching experiment for both soil and water (leachate), the method were validated following the European guidelines on analytical quality control and method validation for pesticide residues in food and feed (SANTE/11,312/2021v2)^24^. There are no need for evaluation of matrix effects because our analysis were by HPLC photodiode array, so there is no signal reduction in this system like LC MS–MS. Controlling our analysis by injection calibration blank, calibration curve, standard check and then free sample blank followed by its spike to check recovery. Spiking samples were performed at three different levels (50, 100 and 200 mg/kg) in free soil and water (leachate) samples. The evaluated parameters included linearity, precision, trueness and limit of quantification (LOQ). Linearity as the deviation of back-calculated values from expected concentrations (± 20%), Precision as the relative standard deviation (RSD, ≤ 20%), Trueness was evaluated based on the average recovery percentages obtained from fortified soil samples (Spike) with acceptable range: 70–120% and LOQ as the lowest validated spike level meeting the acceptance criteria for trueness and precision.

## Results

### Adsorption isotherm experiment of the tested pesticides:

The limits of detection (LOD) for adsorption isotherm method analysis of the tested pesticides were 1.88, 2.13, 1.87, 1.88, 1.99, 1.84, 1.87, 1.98, and 1.79 mg L^-1^ for acetamiprid, difenoconazole, indoxacarb, lufenuron, chlorantraniliprole, metalaxyl, emamectin benzoate, pendimethalin, and tebuconazole, respectively. While, the limits of quantification (LOQ) for this method were 6.26, 7.10, 6.24, 6.26, 6.65, 6.13, 6.25, 6.61, and 5.96 mg L^-1^ for acetamiprid, difenoconazole, indoxacarb, lufenuron, chlorantraniliprole, metalaxyl, emamectin benzoate, pendimethalin, and tebuconazole, respectively. Both limit of detection and limit of quantification were calculated according to Eurachem guide^32^. The optimal wavelength spectrum for each pesticide was selected based on intensity and interference. It also includes the retention times for each pesticide in the adsorption isotherm experiment. See Table 3.

**Table 3:** Optimal wavelengths for adsorption isotherm and leaching experiment.

Pesticides	Wavelengths	Retention time	
		Ads. isotherm	Leaching
Acetamiprid	240 nm	7.62	7.62
Chlorantraniliprole	205 nm	13.27	13.27
Difenoconazole	205 nm	21.50	21.50
Emamectin Benzoate	240 nm	15.23	7.60
Indoxacarb	205 nm	23.56	23.56
Lufenuron	210 nm	25.01	6.30
Metalaxyl	205 nm	11.36	11.36
Pendimethalin	240 nm	26.03	26.03
Tebuconazole	205 nm	16.79	17.20

The Limit of Quantitation (LOQ) for the target analytes was established between 6 and 7 mg/kg, depending on the specific chemical properties of each compound. Method performance was validated through recovery studies at various spiking levels, yielding values between 85 and 109%, which align with international quality standards. Precision was demonstrated by Relative Standard Deviation (RSD) values consistently remaining under 16%, confirming high method reproducibility. Furthermore, the expanded measurement uncertainty at a 95% confidence interval was calculated from the RSD and found to be within ± 50%. To ensure ongoing system stability, daily quality control was performed using spiked samples at 200 mg/kg; all monitoring results fell within the strict acceptance criteria of 70–120% recovery and an RSD of ≤ 20%.

Data in Table 4, presented that the amount of pesticides adsorbed on clay soil at different intervals (Zero to 72 h) for acetamiprid, metalaxyl, and tebuconazole reached equilibrium adsorption isotherm at 24 h. In contrast emamectin benzoate, chlorantraniliprole, difenoconazole, indoxacarb, lufenuron, and pendimethalin reached equilibrium adsorption isotherm at 48 h. See Table 4.

**Table 4:** Equilibrium intervals for adsorption isotherm of the tested pesticides.

Pesticide/Time (mean)	Zero	1 h	6 h	12 h	24 h	48 h	72 h
Acetamiprid	210.0	209.6	206.4	202.6	195.0	196.0	195.0
Emamectin benzoate	201.0	198.0	184.7	172.1	93.3	13.7	12.4
Metalaxyl	208.0	205.0	201.1	196.1	195.7	193.7	194.0
Chlorantraniliprole	197.0	180.0	70.1	55.3	13.0	7.0	< LOQ
Tebuconazole	205.0	189.9	147.6	102.8	96.9	97.3	97.5
Difenoconazole	195.3	111.2	59.7	18.2	7.5	< LOQ	< LOQ
Indoxacarb	191.9	167.8	91.4	21.6	6.5	< LOQ	N.D
Lufenuron	193.0	169.1	94.3	24.7	9.1	< LOQ	N.D
Pendimethalin	189.0	107.9	89.0	19.3	7.0	< LOQ	N.D

*LOQ* Limit of Quantification. *N.D* Not detected.

Data in Table 5, illustrate that acetamiprid and metalaxyl had the lowest amount of pesticides adsorbed on clay soil based on the equilibrium time for each pesticide. Whereas, emamectin benzoate, chlorantraniliprole, difenoconazole, indoxacarb, lufenuron, and pendimethalin showed the highest levels of pesticide adsorbtion on clay soil. See Table 5.

**Table 5:** Pesticides concentrations at the time of equilibrium.

Pesticide/Concentration	Time	100 mg/L	200 mg/L	300 mg/L
Acetamiprid	24 h	97.0	196.0	292.0
Emamectin benzoate	48 h	7.4	12.1	20.1
Metalaxyl	24 h	98.0	197.5	295.0
Chlorantraniliprole	48 h	< LOQ	< LOQ	< LOQ
Tebuconazole	24 h	45.8	94.3	138.0
Difenoconazole	48 h	< LOQ	< LOQ	< LOQ
Indoxacarb	48 h	< LOQ	< LOQ	< LOQ
Lufenuron	48 h	< LOQ	< LOQ	< LOQ
Pendimethalin	48 h	< LOQ	< LOQ	< LOQ

*LOQ* Limit of Quantification.

Acetamiprid and metalaxyl did not line up with the Freundlich model for adsorption isotherm as their correlation coefficient (R^2^) values are less than 0.95. While, the remaining pesticides were consistent with the model. Based on the K_f_ values, tebuconazole, difenoconazole, and emamectin benzoate showed the highest affinity to clay soil, while chlorantraniliprole, lufenuron, and indoxacarb exhibited the lowest attraction. See Table 6.

**Table 6:** Values of R^2^, K_f_ and n according to Freundlich model.

Pesticide	n	K_f_	R^2^
Acetamiprid	0.978	1624	0.854
Emamectin Benzoate	1.14	3.42	0.972
Metalaxyl	0.945	2841	0.812
Chlorantraniliprole	1.01	1.74	0.990
Tebuconazole	0.986	34.1	0.998
Difenoconazole	1.03	5.21	0.985
Indoxacarb	0.876	2.26	0.984
Lufenuron	0.997	1.85	0.988
Pendimethalin	0.855	2.56	0.992

*K*_*f*_ Adsorption capacity; *n* Adsorption intensity; *R*^*2*^ Correlation coefficient.

Similarly, according to Langmuir model, Table 7 indicates that the R^2^ values for acetamiprid and metalaxyl did not match with the Langmuir model for adsorption isotherm as their values are less than 0.950. In contrast, the rest of the pesticides aligned with the Langmuir model. Based on the R_L_ values, tebuconazole showed the most favorable adsorption as they had the highest R_L_ value, close to one due to higher K_L_ mean stronger binding affinity. However, lufenuron, emamectin benzoate, and chlorantraniliprole exhibited moderately favorable adsorption, with R_L_ values ranging from 0.350 to 0.433. Meanwhile, a higher q_max_ indicates better adsorption capacity of the pesticides. See Table 7.

**Table 7:** Values of q_max_, K_L_, R_L_ and R^2^ according to Langmuir model.

Pesticide	q_max_	K_L_	R_L_	R^2^
Acetamiprid	0.371	0.003	0.80	0.846
Emamectin Benzoate	21.8	0.013	0.43	0.975
Metalaxyl	0.194	0.003	0.748	0.79
Chlorantraniliprole	92.6	0.018	0.353	0.993
Tebuconazole	68.5	0.0	0.958	0.998
Difenoconazole	67.6	0.075	0.118	0.989
Indoxacarb	24.3	0.094	0.096	0.989
Lufenuron	137	0.013	0.434	0.992
Pendimethalin	23.6	0.106	0.087	0.994

q_max_: Maximum Adsorption Capacity. K_L_: Langmuir Constant. R_L_: Separation factor. R^2^: Correlation coefficient.

Among the two isotherm models, the R^2^ values were comparable. For the Freundlich model, the highest correlation coefficients (R^2^) were observed for tebuconazole (0.998), pendimethalin (0.992), chlorantraniliprole (0.989), and lufenuron (0.988). Similarly, for the Langmuir model, the highest R^2^ values for these pesticides were 0.998, 0.994, 0.993, and 0.992, respectively.

### The leaching behavior of the tested pesticides

Regarding leaching isotherm experiment, the LOD values for leaching experiment were 1.13, 1.28, 1.12, 1.13, 1.20, 1.13, 1.34, 1.02, and 1.02 mg L^-1^ for acetamiprid, difenoconazole, indoxacarb, lufenuron, chlorantraniliprole, metalaxyl, emamectin benzoate, pendimethalin, and tebuconazole, respectively, while the LOQ for this method were 3.76, 4.26, 3.74, 3.75, 3.99, 3.76, 4.46, 3.39, and 3.39 mg L^-1^ for the same pesticides, respectively. The optimal wavelength spectrum for each pesticide was selected based on intensity and interference. It also includes the retention times for each pesticide in leaching behavior experiment. See Table 3.

The LOQ for all target compounds was from 3.4 to 4.5 mg/kg depends on the compound. Recovery across spiking levels and matrices (Soil and Water) ranged from 75 to 104%, confirming good method performance. Precision was consistently below 19% RSD, demonstrating reproducibility. Measurement uncertainty, expressed as expanded uncertainty at 95% confidence, was within ± 50%, calculated from RSD. Daily monitoring of spiked samples at 100 mg/kg was carried out to verify method stability, with acceptance limits set at 70–120% recovery and ≤ 20% RSD.

Data in Table 8 showed that all leachates (water) from blank samples for all pesticides ranged from not detected (N.D) to below the limit of quantification (< LOQ) by using solid phase extraction (SPE) with polymer based hydrophilic lipophilic balanced SPE cartridges (Oasis HLB)^27–29^. Similarly, all blank soil samples for all pesticides ranged from N.D. to < LOQ. For the treated samples, all leachates containing the tested pesticides were also in the range of N.D. to < LOQ, except for acetamiprid and metalaxyl, which had concentrations of 7.90 mg/L and 19.5 mg/L, corresponding to 2.26% and 15.6% of the fortification concentration, respectively. Data also indicated that the nine investigated pesticides could be classified into three categories according to their water solubility. Highly water-soluble pesticides included acetamiprid and metalaxyl; moderately soluble pesticides comprised emamectin benzoate, tebuconazole, difenoconazole, and indoxacarb; whereas low-solubility pesticides included chlorantraniliprole, lufenuron, and pendimethalin. Within the highly soluble category, the maximum concentration of acetamiprid was detected at a soil depth of 20–30 cm, representing 50% of the total fortified amount, while metalaxyl exhibited its highest concentration (35.5%) at a depth of 30–50 cm. For the moderately soluble group, the maximum concentrations of emamectin benzoate, tebuconazole, difenoconazole, and indoxacarb were 80.2%, 78.8%, 70.9%, and 76.4%, respectively, and were found at a soil depth of Zero – 10 cm. While, the low soluble pesticides, the greatest concentrations of chlorantraniliprole, lefenuron and pendimethain were 93.3%, 87.4% and 98.2% respectively, and were detected at a soil depth of Zero–10 cm. See Table 8. All soil samples in leaching experiment were analyzed by QUECHERS method for pesticide residue analysis according to authors^30,31^

**Table 8:** The pesticide concentrations detected in leachates and soil samples at different depths.

	Pesticides	Fortified concentration (mg/L)	Leachate Avg. Conc. (mg/L)	Soil depth (cm)			
				Avg. concentration (mg/kg)			
				0—10	10—20	20—30	30—50
High soluble pesticides	Acetamiprid	350	7.90	5.0	80.05	174.8	94.3
	Metalaxyl	125	19.5	< LOQ	20.6	36.0	44.4
Moderate soluble pesticides	Emamectin Benzoate	100	N.D	80.2	10.8	9.25	7.96
	Tebuconazole	450	< LOQ	354.5	87.0	19.0	< LOQ
	Difenconazole	400	< LOQ	284	59.4	27.6	21.7
	Indoxacarb	250	N.D	191	53.1	7.0	< LOQ
Low soluble pesticides	Chlorantraniliprole	350	< LOQ	326.7	23.4	6.00	< LOQ
	Lofenuron	250	N.D	218.4	8.95	4.00	< LOQ
	Pendimethalin	450	N.D	422	8.24	< LOQ	< LOQ

*LOQ* Limit of Quantification; *N.D* Not detected.

## Discussion

The fate of pesticides can be represented by a triangle in which one vertex represents the chemical and physical properties of the pesticide (e.g., molecular weight, water solubility, and vapor pressure), considered the only variable, while soil characteristics and environmental factors remain constant under the same conditions.

In the present study, the tested pesticides can be classified based on their water solubility into three groups, highly soluble pesticides (acetamiprid and metalaxyl), moderately soluble pesticides (emamectin benzoate, difenoconazole, indoxacarb and tebuconazole), and low-solubility pesticides (chlorantraniliprole, lufenuron, and pendimethalin). This classification is important because pesticides with high water solubility generally exhibit lower sorption to soil particles and have a greater potential for leaching into groundwater. The Langmuir and Freundlich models are the two most fundamental models used to describe adsorption isotherms. The present study showed that emamectin benzoate, chlorantraniliprole, tebuconazole, difenoconazole, indoxacarb, lufenuron, and pendimethalin fit both the Langmuir and Freundlich adsorption isotherm models; however, all compounds showed higher correlation coefficients with the Langmuir model than with the Freundlich model.

The better fit of the Langmuir adsorption isotherm compared to the Freundlich adsorption isotherm indicates that pesticide adsorption was mainly governed by monolayer, site specific interactions on relatively homogeneous clay surfaces with a finite number of binding sites. The low organic matter content and clay dominated nature of the soil likely reduced surface heterogeneity, favoring Langmuir behavior. Moreover, the predominantly low to moderate solubility and hydrophobic character of most pesticides enhanced strong interactions (e.g., hydrogen bonding and van der Waals forces), resulting in saturation (q_max_) consistent with a site limited mechanism rather than multilayer adsorption. Similar trends have been reported in soil systems where uniform surface energetics promote Langmuir type adsorption Sparks, 2003 and Bennett, 2003^33,34^.

Adsorption of emamectin benzoate in the tested soil was about 93% of the fortified concentration and also had a high adsorption capacity according to the q_max_ value (21.8) in the Langmuir model. The present results are consistent with previous investigations conducted in greenhouse soils^35^. Chlorantraniliprole adsorption in the tested soil accounted for approximately 98.5% of the fortified concentration, which aligns with the findings reported by El-Aswad et al.^36^. The adsorption process demonstrated a high sorption capacity, as indicated by the Langmuir maximum adsorption capacity (q_max_ = 92.6). These observations are in agreement with earlier studies evaluating the adsorption isotherm behavior of chlorantraniliprole in soil systems^4^.

Furthermore, tebuconazole and difenoconazole exhibited adsorption rates of approximately 54.0% and 99.5% in the tested soil, respectively. The adsorption behavior of both fungicides was well described by the Langmuir model, with maximum adsorption capacities (q_max_) of 68.5 for tebuconazole and 67.5 for difenoconazole. This finding agrees with previous research which demonstrated that tebuconazole exhibits a significantly high adsorption affinity in wheat soil^37^. The present results also agree with an earlier study that investigated the adsorption–desorption behavior of difenoconazole and reported high sorption in soil. However, in contrast to the present study, the authors identified the Freundlich model as the most appropriate model to describe the sorption isotherms^38^.

Additionally, indoxacarb and pendimethalin exhibited high adsorption capacities, with Langmuir q_max_ values of 24.3 and 23.6, respectively, indicating a strong affinity for the tested soil. These findings are consistent with Campbell et al.^39^, who reported that indoxacarb showed strong binding across all tested Hawaiian soils, suggesting that both pesticides interact effectively with soil, likely through hydrophobic and van der Waals interactions. These data also align with a previous investigation which examined the adsorption–desorption of pendimethalin in soils with varying organic matter content using the Freundlich model^40^. Regarding lufenuron, it showed very high adsorption in the tested soil, with nearly 98.7% of the fortified concentration retained. The insecticide demonstrated a very high adsorption capacity (Langmuir q_max_ = 137), suggesting strong binding to soil matrix and limited mobility. These findings align with those of Louie-Juzwiak and Waleko^41^, in which lufenuron was found to be strongly adsorbed and mostly immobile in the tested soils, indicating lower potential for leaching or surface runoff.

On the contrary, the present work showed that acetamiprid and metalaxyl did not fit the Langmuir or Freundlich adsorption isotherm models, as their R^2^ values were less than 0.950. Furthermore, the adsorption of acetamiprid and metalaxyl in the tested soil was only about 2.50% and 1.50% of the fortified concentration, respectively. Our results contrast with previous studies^42^, which examined the fate of acetamiprid in sandy loam soils, investigated the adsorption behavior of metalaxyl in soil^43^. This difference may be attributed to variations in soil type and texture, as the soil in the present study was clay, whereas the previous studies were conducted in loamy sandy soils. Research of authors Potts et al.^42^ and his group reported that the highest adsorption of acetamiprid occurred in soils with high organic matter content. This is consistent with our findings, as the soil in the present study contained relatively low organic matter (~ 2.00%) and exhibited low adsorption capacity. The reduced adsorption may also be influenced by the high solubility of both acetamiprid and metalaxyl, which limits their retention in the soil.

The mobility and leaching behavior of the tested pesticides varied considerably. High-leaching pesticides, such as acetamiprid and metalaxyl, exhibited substantial movement in the soil, whereas low-leaching pesticides, including emamectin benzoate, chlorantraniliprole, tebuconazole, difenoconazole, indoxacarb, lufenuron, and pendimethalin, were mostly retained in the upper soil layers. Acetamiprid and metalaxyl demonstrated the greatest leaching potential, with the highest concentrations of the fortified concentration detected in the 20–30 cm soil layer for acetamiprid and in the 30–50 cm layer for metalaxyl. This is consistent with earlier studies tested the leaching of acetamiprid in sandy loam soil^42^, and movement of metalaxyl in soil^43^. Furthermore, these data also agree with our experiments on adsorption isotherm, as acetamiprid and metalaxyl did not exhibit significant adsorption values because of their high solubility. Therefore, these pesticides tend to remain dissolved in water and move with it rather than adsorb onto the soil particles.

The remaining tested pesticides exhibited low leaching and mobility, with the highest concentration of the fortified amount detected in the zero – 10 cm soil layer. Study on the mobility of emamectin benzoate in the soils showed also that the majority of the insecticide was concentrated at the top of the soil column^44,45^. Additionally, our experiment on the leaching behavior of chlorantraniliprole agreed with the other results that classified chlorantraniliprole in clay loam soil as an immobile pesticide^46^. The leaching of tebuconazole and difenoconazole observed in the current research aligns with the findings of Aldana et al.^47^, who examined tebuconazole leaching in Colombian soil and reported the highest concentrations in the top soil layer and agree with studies that revealed lower mobility of difenoconazole in five different soils^12^. All of the above-mentioned pesticides are considered to have low water solubility (< 50 mg/L) and moderate molecular weights (300–500 g/mol). Moreover, most of them contain halogen atoms, such as F, Cl, and Br, as in the cases of lufenuron and indoxacarb. These factors enhance their binding to soil particles, resulting in reduced mobility in soil^48^.

In addition, the leaching behavior of indoxacarb was consistent with the earlier results pointed out that the highest concentration was found within the top 10 cm in sandy loam soil^49^. Moreover, the results of leaching behavior of lufenuron and pendimethalin were consistent with data obtained by Louie-Juzwiak and Waleko^41^, who found that lufenuron tend to be immobile, possessing little potential for leaching and runoff. This also aligns with other results concluded that pendimethalin did not leach below a depth of 10 cm in the sandy clay loamy soil^50^.

Concerning the two highly soluble pesticides; acetamiprid and metalaxyl (based on our classification), both compounds exhibited the highest water solubility and the lowest molecular weights among the nine studied pesticides. These physicochemical properties may explain their poor fit to the adsorption isotherm models, probably due to their greater mobility in the aqueous phase and weaker interaction with soil adsorption sites. Although the two pesticides exhibited the lowest adsorption values, they showed very high leaching potential. The low adsorption and high leaching of acetamiprid (a neonicotinoid pesticide) are due to its high-water solubility and molecular structure, which favors systemic movement within crops. Acetamiprid is a highly polar pesticide (C₁₀H₁₁ClN₄) containing a chloropyridinyl ring and a cyano group, enabling it to easily form hydrogen bonds with water molecules and facilitating its mobility in the soil environment. Additionally, acetamiprid is a non-ionized polar compound at pH 7.00 and lacks a strong positive charge (cation) to bind effectively to the negatively charged clay particles. Soils with a high percent of organic matter and exchangeable cations showed lower desorption and higher adsorption as described by Lalín-Pousa et al.^51^. Metalaxyl, an acylalanine fungicide, is also designed as a systemic pesticide, absorbed by plant roots and translocated through the xylem to all parts of the plant. This requires weak attachment to soil particles and high solubility^52^. Metalaxyl (C₁₅H₂₁NO₄) contains amide functional groups and is uncharged, which contributes to its high leaching and low adsorption, as it cannot form strong ionic bonds with negatively charged clay. It can only weakly interact with soil particles through van der Waals forces. Highly soluble pesticides can move freely through soil pores without strong binding. Although clay has a large surface area and is generally efficient at trapping pesticides, it cannot retain compounds that do not bind effectively^53^.

As for the four moderately soluble pesticides, emamectin benzoate (B1a, C₄₉H₇₅NO₁₃) is a large hydrophobic molecule from the avermectin family. It is a macrocyclic lactone that prefers to bind to soil particles rather than move with water due to its high molecular weight (~ 1008 g/mol), which provides a very large surface area and increases opportunities for van der Waals interactions. Emamectin benzoate also contains several active functional groups, such as NH₂, OH, and COOH. The NH₂ group carries a partial positive charge, and since Egyptian soils are rich in negatively charged clay, strong electrostatic attraction occurs, contributing to the pesticide’s low mobility in soil^44^. The other pesticides in the moderately soluble group, difenoconazole (C₁₉H₁₇Cl₂N₃O₃) and tebuconazole (C₁₆H₂₂ClN₃O) are highly lipophilic due to their multiple aromatic rings, which do not interact favorably with water and instead associate with soil organic carbon and clay surfaces. The specific functional groups in difenoconazole and tebuconazole facilitate soil binding through van der Waals forces, arising from strong dispersion interactions between large carbon rings and chlorine atoms, as well as hydrogen bonding between the nitrogen atoms in the triazole groups and OH groups in organic matter and clay minerals^54^. For the last member in this group, indoxacarb (C₂₂H₁₇ClF₃N₃O₇), an oxadiazine insecticide with low water solubility, is chemically designed to be lipophilic and resistant to movement through soil. Its structure, which includes two chlorine atoms and a trifluoromethoxy group, increases both its molecular weight and hydrophobicity, and also generating strong dipole interactions. Although indoxacarb has a high affinity for organic matter, it also exhibits strong adsorption to clay. This is facilitated by the oxygen and nitrogen atoms in its oxadiazine ring, which can form hydrogen bonds with silanol and aluminol groups on clay mineral surfaces^55^.

The three low-soluble pesticides, chlorantraniliprole (C₁₈H₁₄BrCl₂N₅O₂), belonging to the anthranilic diamide class of insecticides, has a large molecular size and specific hydrophobic functional groups due to the presence of halogen atoms (one Br and two Cl), which increase its lipophilicity and molecular viscosity through van der Waals interactions. Because chlorantraniliprole has low solubility and relatively high partition coefficient (Koc), low leaching and greater soil surface persistence in soil were observed. Additionally, the insecticide contains amide groups, allowing it to form strong hydrogen bonds with OH groups present in soil organic matter and on the surface of clay minerals^56^.

Lufenuron (C₁₇H₈Cl₂F₈N₂O₃) is a water-insoluble benzoylurea insecticide (insect growth regulator). Its chemical structure contains eight fluorine atoms and two chlorine atoms, which increase its molecular weight and lipophilicity. The presence of these ten halogen atoms enhances van der Waals forces, effectively binding the pesticide to clay surfaces and soil organic matter. The binding of lufenuron is not limited to simple surface adsorption; its noticeable hydrophobicity likely facilitates the formation of condensed or multi-layer phases on soil particles, reducing interactive with the aqueous phase^56^. Pendimethalin (C₁₃H₁₉N₃O₄), a dinitroaniline herbicide, exhibits even stronger soil affinity. While some pesticides may interact with the water phase, pendimethalin is highly retained by the soil and is nearly immobile due to its high log P value of 5.40, the highest among the nine tested pesticides. Its chemical structure includes a benzene ring substituted with two nitro groups, which are strong electron-withdrawing groups. This group creates a specific electronic distribution, enabling pendimethalin to form charge-transfer complexes with clay mineral surfaces and humic acids. Even though the tested soil has low organic matter (~ 2.00%), the high clay content provides a large surface area, and the presence of multiple functional atoms in these pesticides facilitates strong interactions with the negatively charged clay surfaces^57^.

## Conclusion

The complexity of pesticide–soil interactions often exceeds the capacity of a single equilibrium model due to soil heterogeneity, degradation, and simultaneous sorption mechanisms. While kinetic models can offer deeper insights into leaching, our findings demonstrate that adsorption and mobility profiles align consistently with the physicochemical properties of tested pesticides.

Small, highly water soluble molecules like metalaxyl and acetamiprid exhibit minimal adsorption and high mobility, posing a significant threat to groundwater. In contrast, complex, high molecular weight compounds such as emamectin benzoate, lufenuron, and pendimethalin bind strongly to clay particles and remain sequestered in the topsoil. Understanding these properties allows for the prediction of environmental behavior, providing a foundation for sustainable pesticide application and the protection of water resources in agricultural regions.

## Data Availability

The datasets used and/or analyzed during the current study available from the corresponding author on reasonable request.
